# Pharmacokinetics of intravenous oxycodone hydrochloride in perioperative patients with liver cancer

**DOI:** 10.3389/fmed.2026.1834903

**Published:** 2026-05-28

**Authors:** Shuwei Shi, Ying Zheng, Yong Wang, Shengchao Li, Rui Feng, Luya Li

**Affiliations:** 1 Pharmacy Department, The Fourth Hospital of Hebei Medical University, Shijiazhuang, China; 2 Hebei Key Laboratory of Clinical Pharmacy, Shijiazhuang, China; 3 Department of Anesthesiology, The Fourth Hospital of Hebei Medical University, Shijiazhuang, China; 4 Department of Hepatobiliary Surgery, The Fourth Hospital of Hebei Medical University, Shijiazhuang, China; 5 School of Disaster and Emergency Medicine, Tianjin University, Tianjin, China

**Keywords:** oxycodone, noroxycodone, liver cancer, HPLC-MS/MS, pharmacokinetics

## Abstract

**Introduction:**

Effective perioperative analgesia in liver cancer patients presents an ongoing clinical challenge. This study investigates oxycodone’s pharmacokinetics in perioperative liver cancer patients with normal liver function to support individualized analgesia.

**Methods:**

This study developed and validated a reliable high-performance liquid chromatography-tandem mass spectrometry (HPLC-MS/MS) method for the simultaneous quantification of oxycodone and its metabolite noroxycodone in human plasma. The method was successfully applied to characterize the pharmacokinetics in nine surgical liver cancer patients following intravenous oxycodone administration, using non-compartmental analysis (NCA).

**Results:**

The developed method showed satisfactory linearity and met validation criteria. In perioperative liver cancer patients, oxycodone exhibited reduced clearance, prolonged half-life, increased volume of distribution, and elevated exposure compared with healthy volunteers. Its metabolite noroxycodone displayed a biphasic profile with consistently higher concentrations in males. Significant sex-related differences were also observed for oxycodone’s area under the plasma concentration-time curve (AUC) and mean residence time (MRT).

**Discussion:**

This study reveals that compared with healthy volunteers, perioperative liver cancer patients with normal liver function exhibit significantly altered oxycodone pharmacokinetics, including reduced clearance, prolonged half-life, increased exposure and volume of distribution, with notable sex differences. These findings support the need for dose reduction, extended monitoring, and individualized analgesic strategies.

## 1 Introduction

Cancer constitutes a substantial portion of the global disease burden and, fueled by persistently increasing incidence and mortality rates, has emerged as a leading cause of death in China since 2010 (1). Pain is among the most common symptoms experienced by cancer patients, rendering effective analgesia an indispensable element of comprehensive cancer care. Opioids play a crucial role in this context. As a representative dual *μ*- and *κ*-opioid receptor agonist, oxycodone is widely used for managing cancer pain and postoperative analgesia (2–4).

**Figure 1:**
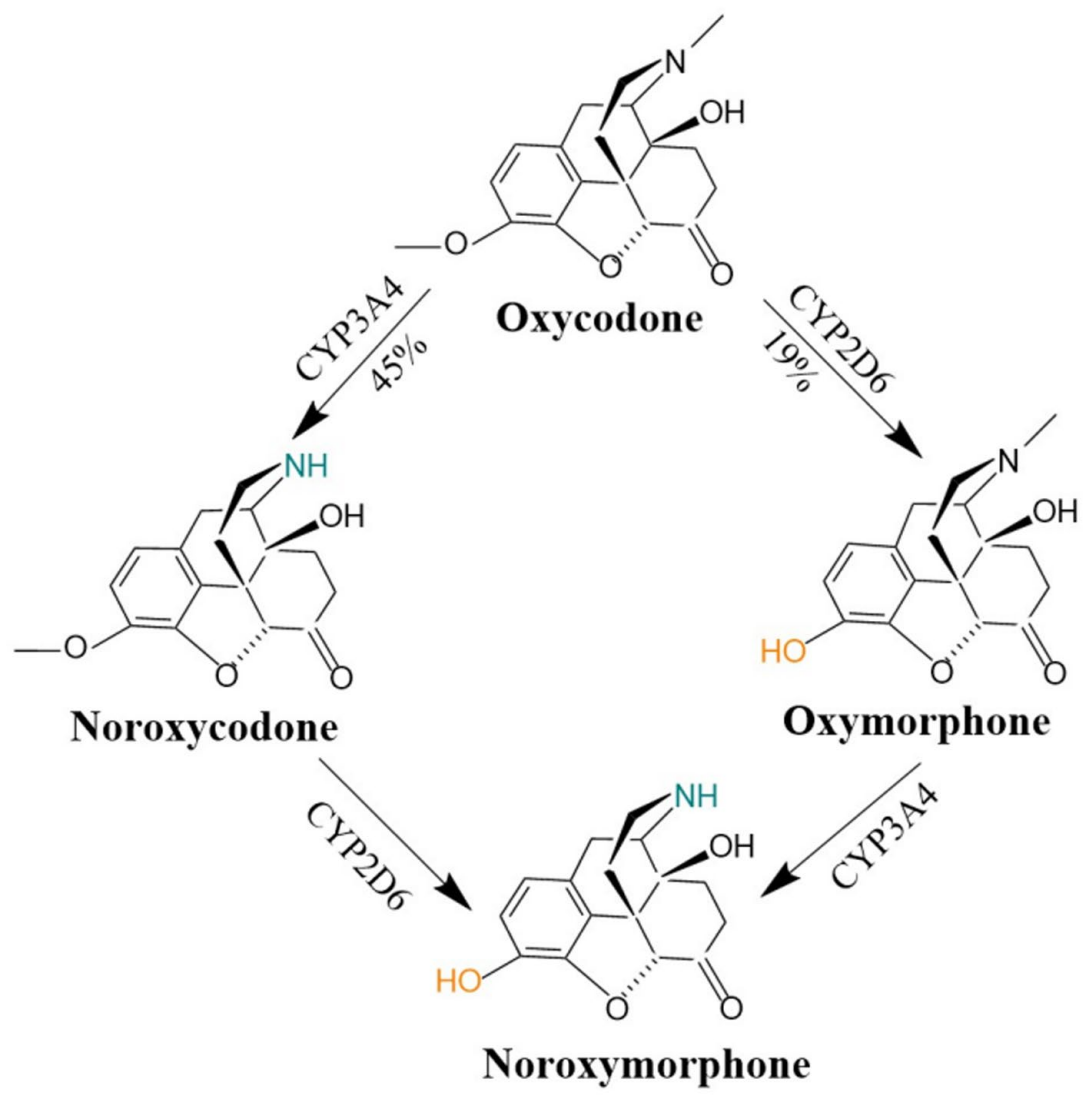
Main metabolic processes of oxycodone *in vivo*.

In humans, oxycodone is primarily metabolized by the hepatic cytochrome P450 (CYP450) enzyme system: approximately 45% of the dose is metabolized via CYP3A4 to noroxycodone, and 19% via CYP2D6 to oxymorphone (Figure 1). Although noroxycodone exhibits lower analgesic efficacy than oxycodone and possesses limited ability to cross the blood–brain barrier, its high plasma concentration and measurable *μ*-opioid receptor affinity suggest the potential for peripherally mediated adverse effects under specific conditions (e.g., drug accumulation) (5–7). Furthermore, genetic polymorphisms in CYP450 enzymes (e.g., CYP2D6) and the concomitant use of CYP450 inhibitors or inducers may lead to significant interindividual variability in both pharmacokinetics and analgesic response (5, 8). Therefore, characterizing the PK profiles of oxycodone and its major metabolites across populations is critical for precision analgesic therapy.

In addition to genetic and drug-related factors, disease-induced pathophysiological alterations can profoundly affect drug metabolism and elimination. This issue is particularly prominent in perioperative patients with hepatocellular carcinoma (HCC). Even when preoperative liver function test results are normal, HCC patients frequently develop perioperative physiological changes, including transient hepatic dysfunction, hypoalbuminemia, ascites, or edema (9, 10). These pathophysiological alterations have been reported to significantly impair drug metabolism and clearance. However, a research gap remains: how do the PK parameters of oxycodone and its metabolite noroxycodone change in the surgical HCC population with baseline normal liver function yet experiencing the aforementioned perioperative physiological perturbations? In the absence of these data, clinicians encounter substantial challenges in achieving scientific and safe individualized dosing.

Therefore, the present study was designed to investigate the PK characteristics of intravenous oxycodone hydrochloride injection in perioperative HCC patients with normal liver function and to elucidate the metabolic profiles of oxycodone and its major metabolite noroxycodone in this patient population. This research aims to provide evidence-based references for optimizing the clinical application of oxycodone hydrochloride injection, thereby promoting its more scientific, rational, and precise use in clinical practice.

## 2 Materials and methods

### 2.1 Drugs and reagents

The drugs used in this study were supplied by the following pharmaceutical manufacturers in China: oxycodone hydrochloride injection (Jiangsu Nhwa Pharmaceutical Co., Ltd.), propofol emulsion injection (Guorui Pharma Co., Ltd.), cisatracurium besylate injection (Hangzhou Ausia Biological Technology Co., Ltd.), and remifentanil hydrochloride for injection (Yichang Humanwell Pharmaceutical Co., Ltd.). Oxycodone and noroxycodone reference standards were purchased from Shanghai Yuanye Bio-Technology Co., Ltd. (China). Papaverine was provided by the National Institutes for Food and Drug Control (China). HPLC-grade methanol and acetonitrile were obtained from Merck KGaA (Germany). Formic acid and ammonium acetate were acquired from Shanghai Aladdin Biochemical Technology Co., Ltd. (China). Ultra-pure water was prepared using a Milli-Q water purification system (Millipore, United States).

### 2.2 Instruments and analytical conditions

The Shimadzu Nexera HPLC system consisted of two LC-40 AD pumps, a CTO-30AS column oven, and a SIL-30 AC autosampler. Chromatographic separation was achieved on a Phenomenex Kinetex F5 column (3.0 × 100 mm, 2.6 μm) maintained at 40.0 °C. The mobile phase was composed of 2 mM ammonium acetate in ultrapure water (with 0.1% v/v formic acid; A) and HPLC-grade methanol (with 0.1% v/v formic acid; B). Chromatographic separation was performed using the gradient elution profile specified in Table 1.

**Table 1:** Gradient elution: composition of mobile phase in liquid chromatography process.

Time(min)	Mobile phase A	Mobile phase B
0.00	30	70
1.50	5	95
2.00	5	95
2.01	30	70
4.00	30	70

MS analysis was performed using an AB Sciex Triple Quad™ 6,500 mass spectrometer (6,500 Q-trap, Applied Biosystems Inc., United States) equipped with an electrospray ionization (ESI) source. The system was operated in positive ion mode, with instrument control and data acquisition managed by Analyst® software.

**Figure 2:**
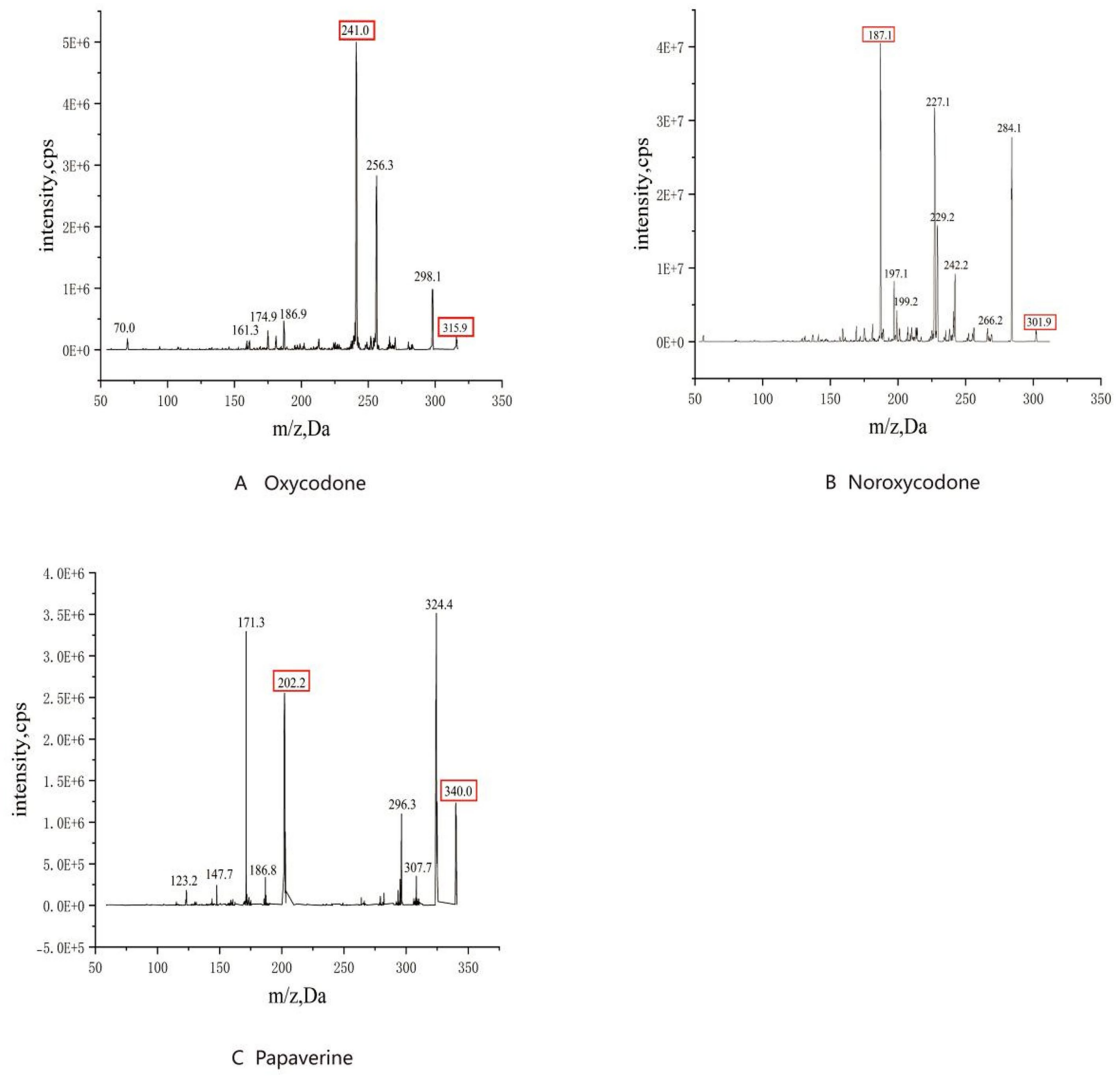
MS/MS spectra of oxycodone, noroxycodone, and the IS (papaverine).

For each analyte, MS/MS transitions were systematically optimized. The final selected multiple reaction monitoring (MRM) transitions and corresponding collision energy parameters are detailed in Table 2. The primary MRM transition for each analyte was used for quantification purposes, while secondary transitions served as qualifier ions. Representative product ion spectra are presented in Figure 2.

**Table 2:** HPLC-MS/MS multi reaction monitoring data of oxycodone, noroxycodone, and papaverine.

Analyte	MRM transition m/z(Q1 > Q3)	CE(V)	DP(V)	EP(V)	CXP(V)
Oxycodone	315.9 > 241.0	38	76	10	21
	315.9 > 256.3	35	76	10	14
Noroxycodone	301.9 > 187.1	34	61	5	9
	301.9 > 284.1	20	70	5	14
	301.9 > 227.1	40	72	10	5
Papaverine	340.0 > 202.2	37	103	10	17
	340.0 > 171.3	49	140	10	15

### 2.3 Preparation of calibration standards and quality control samples

Two independent stock solutions for oxycodone and noroxycodone at a concentration of 1.0 mg/mL in methanol were used to prepare the calibration curve and quality control (QC) samples, respectively. A stock solution of IS was also made in methanol at a concentration of 1.0 mg/mL. All of these solutions were stored in the refrigerator at −80 °C before use. The work solutions of oxycodone and noroxycodone were freshly prepared by further diluting the stock solutions with methanol–water (1:1, v/v). The stock solution of IS was diluted with methanol to obtain final concentrations of 15 ng/mL as precipitant solvent for plasma.

Calibration standards for plasma were prepared by spiking 10 μL of the corresponding work solutions into 190 μL of blank human plasma to obtain the final concentrations of 0.5, 1.5, 5.0, 20.0, 50.0, 100.0 and 200 ng/mL for oxycodone and 0.7, 1.4, 5.0, 10.0, 20.0, 50.0 and 100.0 ng/mL for noroxycodone. QC solutions for oxycodone and noroxycodone including the lower limit of quantitation (LLOQ; 0.5 and 0.7 ng/mL), low quality control (LQC; 1.25 and 2.0 ng/mL), middle quality control (MQC; 25.0 and 15.0 ng/mL), and high quality control (HQC; 150.0 and 75.0 ng/mL) were prepared in the same way.

### 2.4 Sample preparation

The protein precipitation method (PPT) was applied to extract the analytes in plasma. To a 55 μL aliquot of the plasma sample, 220 μL precipitant solvent containing 15 ng/mL IS in acetonitrile was added. The mixture was vortex-mixed for 2 min and centrifuged at 3285 g for 15 min at 4 °C. Then 110 μL of the supernatant was transferred into 40 μL water followed by vortex-mixing for 5 min. Finally, 2 μL of the supernatant was injected into the HPLC-MS/MS system for analysis.

### 2.5 Method validation

Full validation of the proposed method was conducted in accordance with the US Food and Drug Administration (FDA) guidelines and the Chinese Pharmacopeia on bioanalytical method validation.

#### 2.5.1 Specificity and selectivity

The selectivity of the method was assessed by comparing the chromatograms of blank plasma samples from six different sources with the corresponding spiked plasma containing oxycodone, noroxycodone, and IS. These samples should be free from interference of the analyte (a response < 20% of LLOQ for oxycodone and noroxycodone and < 5% for the IS) at the retention time.

#### 2.5.2 Carryover

Blank plasma samples were injected immediately following the upper limit of quantification (ULOQ) sample to assess potential carryover effects.

#### 2.5.3 LLOQ, calibration curve and linearity

The calibration curve was constructed at seven concentration levels (0.5–200 ng/mL for oxycodone and 0.7–100 ng/mL for noroxycodone). The regression equation was calculated using the concentration of the analyte (oxycodone or noroxycodone) as the independent variable (x), the peak area ratio of the analyte to IS as the dependent variable (y), and 1/x^2^ as the weighting factor. The regression coefficient (r^2^) was required to be > 0.99 to ensure linearity. LLOQ was defined as the lowest concentration of the calibration curve with S/N ≥ 10. The accuracy was within 80–120% for LLOQ and 85–115% for other samples.

#### 2.5.4 Precision and accuracy

Intra-batch and inter-batch precision and accuracy were evaluated by determining the QC samples of oxycodone and noroxycodone at four concentration levels in six replicates on the same day and on three separate days, respectively. The precision was calculated using the relative standard deviation (RSD) as follows: RSD (%) = (SD / mean) × 100%, where SD is the standard deviation of the measured concentrations and mean is the mean concentration. Accuracy was calculated as (measured concentration / theoretical concentration) × 100%. The concentration of each sample was determined using the prepared calibration curve and was analyzed in the same batch. Intra-batch and inter-batch variations of the QC samples in the developed method were <15% (±20% at LLOQ) for all analytes.

#### 2.5.5 Dilution study

Standard samples containing oxycodone 500 ng/mL and noroxycodone 200 ng/mL were prepared separately and then diluted 5-fold with blank plasma. Six parallel operations were performed. The mean accuracy of the diluted quality controls was required to be within ±15% of the labeled value, with precision also within ±15%. This indicates that the accuracy and precision of this method were not affected by plasma samples diluted with a blank matrix.

#### 2.5.6 Matrix effect and recovery

The IS-normalized matrix factor (MF) was calculated by the ratio of the peak area in the presence of the matrix (blank matrix spiked post-extraction) to the peak area in the absence of the matrix (aqueous solution) for LQC, MQC, and HQC samples. The extraction recovery was calculated by the ratio of the peak area from the matrix (blank matrix spiked pre-extraction) to the peak area in the presence of the matrix (blank matrix spiked post-extraction).

#### 2.5.7 Stability

The stability was evaluated by analyzing five replicates of low, medium, and high concentration stability quality control samples under the following storage conditions: freeze–thaw stability (stored at −80 °C for 24 h and thawed to room temperature for three cycles); short-term stability (samples kept at room temperature for at least 24 h); long-term stability (samples stored at −80 °C for 60 days) and autosampler stability (samples placed in the autosampler at room temperature for 12 h before analysis).

### 2.6 Patients and anesthesia methods

#### 2.6.1 Patients

All patients provided signed informed consent prior to anesthesia administration. This study was approved by the Ethics Committee of the Fourth Hospital of Hebei Medical University (Approval No.: 2022065) and was conducted in accordance with the principles of the Declaration of Helsinki. The inclusion criteria were as follows: (1) meeting the diagnostic criteria for HCC as outlined in the “Standard for Diagnosis and Treatment of Primary Liver Cancer (2019 Edition)” (11); (2) willingness to undergo surgical treatment; (3) Child-Pugh classification (CTP score) of Grade A; (4) American Society of Anesthesiologists (ASA) physical status classification of 1 or 2; (5) patient’s body weight within 30% of their ideal body weight. The exclusion criteria were as follows: (1) Allergy to oxycodone; (2) Pregnancy; (3) Long-term use of opioid medications; (4) Excessive intraoperative blood loss; (5) Chronic kidney disease or severe pulmonary disease; (6) History of chronic pain.

#### 2.6.2 Anesthesia methods

All patients were routinely fasted for 6–8 h before surgery. Upon entering the operating room, all patients underwent radial artery puncture under local anesthesia with lidocaine to monitor mean arterial pressure. A peripheral intravenous line was established on the left side for drug administration and on the right side for blood sampling. Monitoring included electrocardiogram (ECG), pulse, oxygen saturation, blood pressure, and bispectral index (BIS). (1) Anesthesia Induction: Oxycodone hydrochloride injection was intravenously administered at a dose of 0.1 mg/kg, propofol emulsion injection at 2 mg/kg, and cisatracurium besylate injection at 0.2 mg/kg. After the patient had completely lost consciousness, endotracheal intubation was performed, followed by mechanical ventilation with a tidal volume of 8–10 mL/kg and a respiratory rate of 12–14 breaths/min. End-tidal CO_2_ was maintained at 35–40 mmHg during the surgery. (2) Anesthesia Maintenance: Remifentanil hydrochloride for injection was continuously infused during the surgery. Intermittent bolus injections of cisatracurium besylate and sevoflurane were administered to keep the BIS between 45 and 60. Fluid management was guided by goal-directed fluid therapy to maintain hemodynamic stability.

#### 2.6.3 Sample collection

Before the intravenous injection of oxycodone hydrochloride injection (0 h) and at 0.083, 0.25, 0.5, 1, 2, 4, 8, 12, and 24 h after the injection, 3 mL of venous blood samples were collected from the contralateral arm and placed into blood collection tubes containing 3.2% sodium citrate. The blood collection tubes containing 3.2% sodium citrate were centrifuged at 1912 g for 10 min. The separated plasma samples were stored at −80 °C in sample and backup tubes for subsequent analysis.

#### 2.6.4 Data analysis

PK parameters were analyzed using WinNonlin (version 8.3) software, and the analysis was based on NCA. Analyte concentrations below the lower limit of quantification were set to 0. Plasma concentrations of oxycodone and noroxycodone from the subjects were collected, and the plasma concentration-time data were fitted to determine PK parameters such as AUC, terminal half-life (T_1/2_), total body clearance (CL), MRT, volume of distribution at steady state (V_ss_), maximum plasma concentration (C_max_), and time to reach Cmax (T_max_).

Plasma concentration-time curves were plotted using GraphPad Prism (version 10.1.2) software. Statistical analysis was performed using SPSS (version 26.0). *p* < 0.05 was considered statistically significant, *p* < 0.01 as highly statistically significant, and *p* < 0.001 as extremely statistically significant. Quantitative data were tested for normal distribution using the Shapiro–Wilk test in SPSS. Quantitative data were expressed as mean ± standard deviation or median. Data following a normal distribution were analyzed using the t-test, while data not following a normal distribution were analyzed using the Mann–Whitney U test (non-parametric test).

## 3 Results

### 3.1 Methodology validation

#### 3.1.1 Specificity

**Figure 3:**
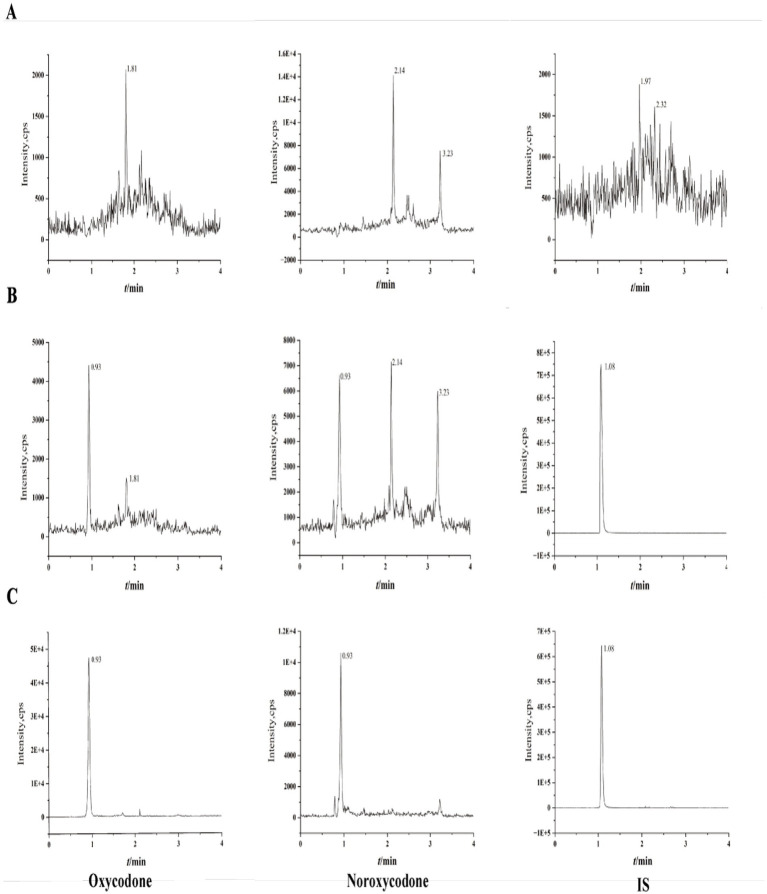
Typical MRM chromatograms of oxycodone (left), noroxycodone (middle) and IS (right). **(A)** blank plasma sample; **(B)** blank plasma sample spiked with oxycodone at 0.5 ng/mL, noroxycodone at 0.7 ng/mL, and IS at 15.0 ng/mL; **(C)** plasma sample from a patient 4 h after intravenous injection of oxycodone (6.6 mg).

The observed retention times for oxycodone, noroxycodone, and the internal standard were 0.93, 0.93, and 1.08 min, respectively. Chromatographic analysis revealed no interfering peaks at these respective retention times. These findings demonstrate the high specificity of the developed method, confirming the absence of interference from plasma endogenous substances on either the target analytes or the internal standard. Representative chromatograms are presented in Figure 3.

#### 3.1.2 Carryover

The peak areas of oxycodone and noroxycodone in blank plasma samples were 6.08–9.35% and 2.68–5.55% of the LLOQ, respectively. The IS responses in all valid batches were nearly negligible, meeting the requirements for clinical sample analysis.

#### 3.1.3 LLOQ, calibration curve and linearity

The standard calibration curves were defined by the equations: y = 0.0093x + 0.0091 (r^2^ = 0.9988) for oxycodone, and y = 0.0045x + 0.0019 (r^2^ = 0.9996) for noroxycodone. All calculated concentrations of calibration standards fell within ±15% of their nominal values, while the LLOQ met the ±20% acceptance criterion. The validated LLOQs were established at 0.5 ng/mL for oxycodone and 0.7 ng/mL for noroxycodone.

#### 3.1.4 Precision and accuracy

The RSD for oxycodone and noroxycodone quality control samples was observed to be below 12% at all QC levels, with accuracy values ranging from 94.1 to 112.8%. As shown in Table 3, the method exhibits excellent reproducibility, thereby confirming its suitability for bioanalytical applications.

**Table 3:** Accuracy and precision of oxycodone and noroxycodone in human plasma by HPLC-MS/MS.

**Compound**	Intra-batch (*n* = 6)				Inter-batch (*n* = 18)		
	QC (nominal conc.) (ng/mL)	Measured conc. (ng/mL, $\bar{x}\pm s$ )	RSD (%)	RE (%)	Measured conc. (ng/mL, $\bar{x}\pm s$ )	RSD (%)	RE (%)
Oxycodone	0.5	0.49 ± 0.04	9.0	−1.7	0.48 ± 0.04	8.5	3.9
	1.25	1.29 ± 0.06	4.9	8.7	1.31 ± 0.06	4.8	4.4
	25.0	27.03 ± 1.77	6.5	8.1	27.54 ± 2.00	7.3	10.2
	150.0	168.61 ± 2.00	1.2	12.4	168.27 ± 4.71	2.8	12.2
Noroxycodone	0.7	0.74 ± 0.04	5.6	5.2	0.75 ± 0.05	6.6	6.6
	2.0	1.97 ± 0.22	11.3	−1.3	1.88 ± 0.17	8.9	−6.2
	15.0	15.97 ± 0.90	5.6	6.5	15.72 ± 0.87	5.5	4.7
	75.0	78.20 ± 3.50	4.2	4.3	78.06 ± 1.97	2.5	4.1

#### 3.1.5 Dilution study

Dilution integrity was assessed by a 5-fold dilution with blank plasma. The mean accuracy values for oxycodone and noroxycodone were 111.0 and 110.0%, respectively, and the precision RSDs were 2.6 and 2.1%, both within acceptable criteria. This confirms that clinical samples can be accurately diluted without impacting the method’s performance.

#### 3.1.6 Matrix effect and recovery

To compensate for potential interference from the endogenous matrix when quantifying oxycodone and noroxycodone, papaverine was employed as the IS in this assay. The matrix effect, corrected by the IS, ranged from ±12% (RSD 3.6–6.3%) for oxycodone and from ±9% (RSD 7.1–10.0%) for noroxycodone. The recovery of the IS was 97.0%. All these parameters being within the predefined ±15% limit validates that the sample preparation process is robust, with no substantial loss of either the analytes or the IS.

#### 3.1.7 Stability

The stability of oxycodone and noroxycodone under the tested conditions was confirmed, with detailed results presented in Table 4.

**Table 4:** Experimental data on the stability of oxycodone and noroxycodone in plasma samples.

Stability experiment	**Compound**	QC (nominal conc.) (ng/mL)	Measured conc.(ng/mL)	RSD (%)	RE (%)
Bench top (4 h at room temperature)	Oxycodone	1.25	1.29	3.9	3.2
		25.0	26.52	5.6	6.1
		150.0	154.21	6.5	2.8
	Noroxycodone	2.0	1.87	7.9	−6.5
		15.0	16.23	7.1	8.2
		75.0	76.80	8.4	2.4
Long-term −80 °C for 2 months	Oxycodone	1.25	1.37	9.6	9.6
		25.0	26.20	5.9	4.8
		150.0	159.30	5.3	6.2
	Noroxycodone	2.0	2.06	4.6	3.0
		15.0	15.58	6.9	3.9
		75.0	76.57	6.7	2.1
Freeze and thaw at −80 °C storage (three cycles)	Oxycodone	1.25	1.31	8.1	4.8
		25.0	26.13	3.1	4.5
		150.0	146.12	8.5	−2.6
	Noroxycodone	2.0	2.06	3.9	3.0
		15.0	15.84	5.1	5.6
		75.0	82.05	7.3	9.4
Auto-sampler (12 h)	Oxycodone	1.25	1.34	6.7	7.2
		25.0	26.98	3.8	7.9
		150.0	154.05	5.4	2.7
	Noroxycodone	2.0	2.21	2.9	10.5
		15.0	15.68	4.8	4.5
		75.0	77.93	6.3	3.9

### 3.2 Patient characteristics

Patient screening was conducted from August to November 2024. Of the 12 individuals screened, three were excluded (two due to lost blood collection sites and one because of excessive blood loss), resulting in a final cohort of nine patients. All subjects demonstrated normal values for alanine aminotransferase (ALT), aspartate transaminase (AST), and total bilirubin (BIL). The baseline demographics and surgical characteristics of the enrolled patients are detailed in Tables 5, 6, respectively.

**Table 5:** Basic information of different patient groups.

Index	All patients (*n* = 9)	Male (*n* = 5)	Female (*n* = 4)
Age (years)	61.22 ± 8.13	65.20 ± 3.96	56.25 ± 9.81
Body height (cm)	164.77 ± 6.66	169.4 ± 4.87	159.00 ± 2.58
Weight (kg)	65.0(60–70)	63.0(60–70)	65.5 (60–67)
BMI (Kg/m^2^)	22.86 (21.79–27.12)	22.32 (21.79–22.86)	26.11 (22.86–27.12)
ALT (U/L)	17.6 (8.0–41.4)	17.6 (9.2–40.8)	20.2 (8.0–41.4)
AST (U/L)	23.4 (10.8–36.8)	23.1 (14.1–34.5)	24.1 (10.8–36.8)
BIL (μmol/L)	15.44 (4.45–26.07)	21.17 (4.45–26.07)	13.30 (7.76–15.63)

No significant differences were observed between the male and female groups.

**Table 6:** Surgery-related information of different groups of patients.

Index	All patients (*n* = 9)	Male (*n* = 5)	Female (*n* = 4)
Hourly fluid volume infused (mL/h)	616.02 ± 142.43	647.27 ± 183.14	576.98 ± 75.66
Hourly urine output (mL/h)	100.04 ± 23.64	101.61 ± 67.15	96.66 ± 30.83
Blood loss (mL)	400 (100–800)	200 (100–600)	500 (100–800)

No significant differences were observed between the male and female groups.

### 3.3 Pharmacokinetics

**Figure 4:**
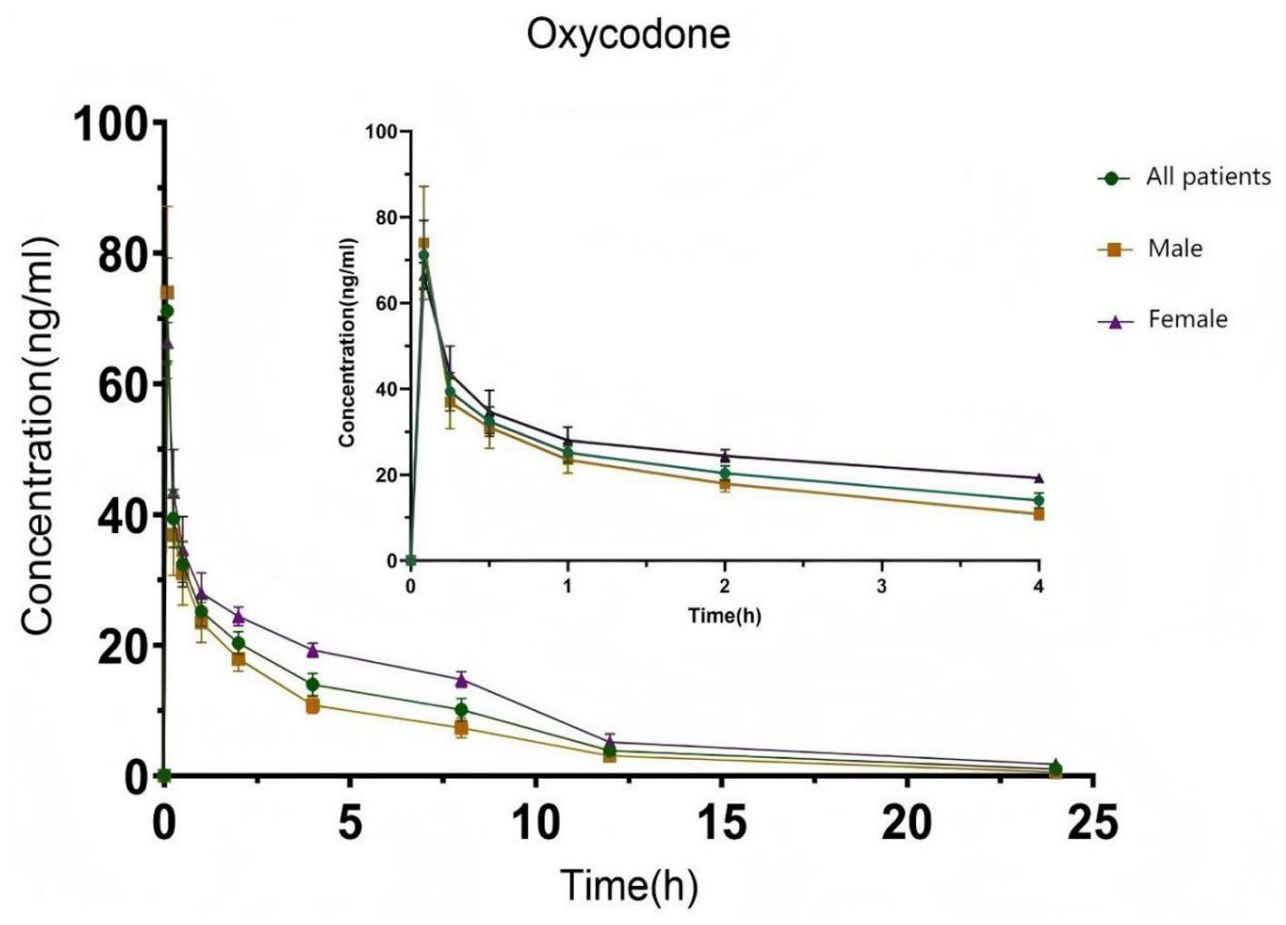
Oxycodone plasma concentration-time curve.

**Figure 5:**
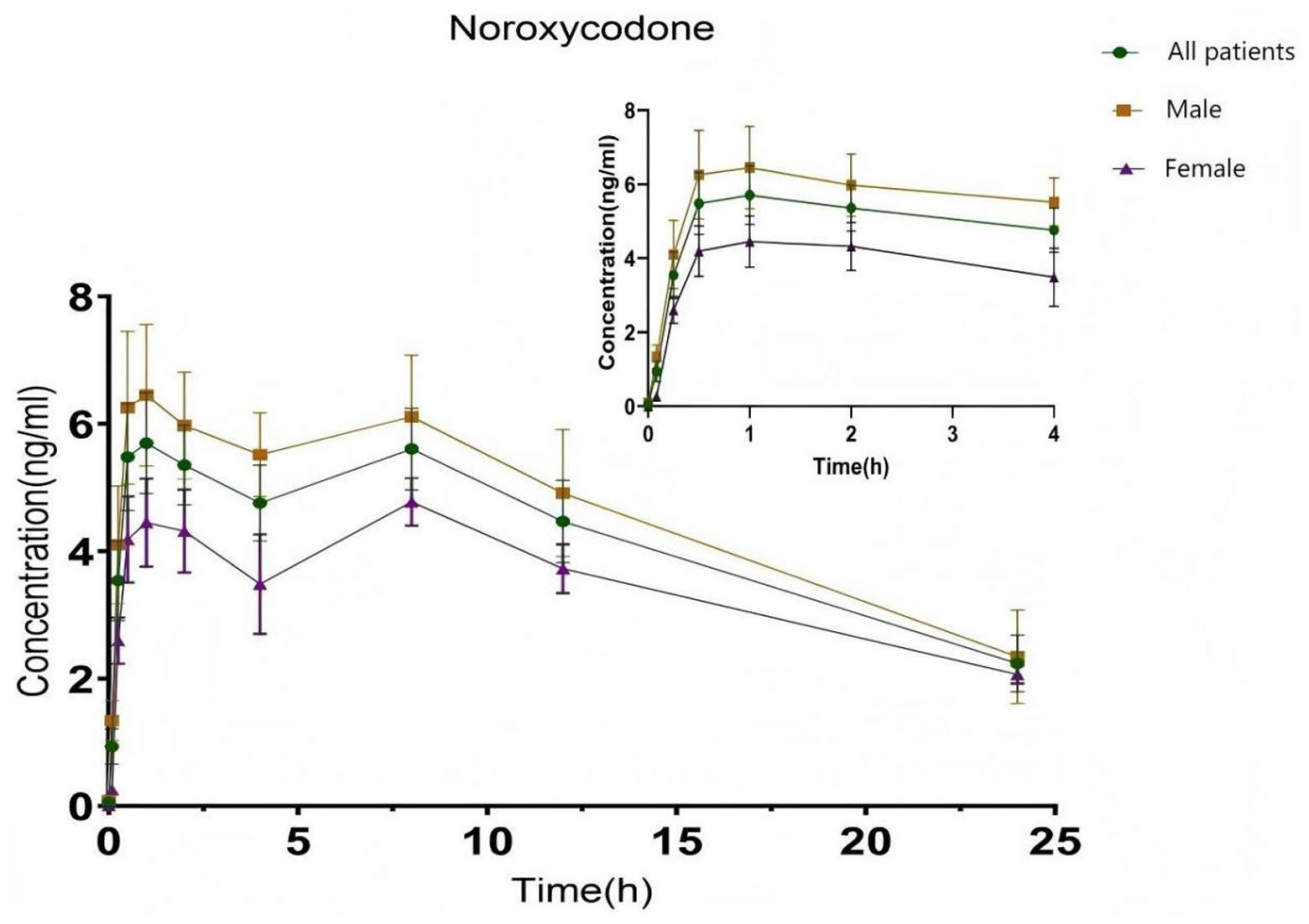
Noroxycodone plasma concentration-time curve.

The mean plasma concentration-time curves of oxycodone and noroxycodone are presented in Figures 4, 5, respectively. The primary PK parameters for both analytes in the nine patients who received intravenous oxycodone hydrochloride injection are summarized in Table 7 (Oxycodone) and Table 8 (Noroxycodone). The results showed that in perioperative liver cancer patients, oxycodone had a C_max_ of 71.14 ng/mL, a Vss of 223.07 L, a T_1/2_ of 4.8 h, an AUC_(0 → ∞)_ of 205.59 h·ng/mL, and a CL of 38.03 L/h. For noroxycodone, the parameters were a Vss of 1026.80 L, dual C_max_ values of 5.92 ng/mL and 5.79 ng/mL observed at T_max_ values of 1.06 h and 7.00 h, respectively, an AUC_(0 → ∞)_ of 145.73 h·ng/mL, and a CL of 60.63 L/h. Gender-related differences were observed for oxycodone’s AUC _(0 → 24h)_, AUC_(0 → ∞)_, MRT_(0 → 24h)_, and MRT_(0 → ∞)_. Furthermore, the plasma concentration of noroxycodone was higher in male patients than in female patients. The extended presence of noroxycodone in the body, characterized by a long retention time, exhibited a biphasic metabolic profile.

**Table 7:** Main PK parameters of oxycodone (*n* = 9, mean ± SD).

Index	Unit	All patients	Male	Female
AUC_(0 → 24h)_	h·ng/mL	198.31 ± 64.71	163.59 ± 61.56	256.85 ± 15.56*
AUC_(0 → ∞)_	h·ng/mL	205.59 ± 69.56	167.08 ± 58.40	269.76 ± 15.22*
T_1/2_	h	4.8 ± 0.95	4.32 ± 0.87	5.60 ± 0.32
C_max_	ng/mL	71.14 ± 22.76	73.97 ± 29.44	66.42 ± 5.15
T_max_	h	0.083	0.083	0.083
CL	L/h	38.03 ± 14.31	42.91 ± 16.52	29.90 ± 3.52
V_d_	L	250.75 ± 59.56	256.58 ± 76.53	241.05 ± 21.83
MRT_(0 → 24h)_	h	5.36 ± 0.80	4.93 ± 0.67	6.08 ± 0.37*
MRT_(0 → ∞)_	h	6.16 ± 1.26	5.47 ± 1.02	7.32 ± 0.54*
V_ss_	L	223.07 ± 53.94	225.8 ± 69.61	218.51 ± 21.12

Comparison of PK parameters between males and females. *p < 0.05 indicates a statistically significant difference.

**Table 8:** Main PK parameters of noroxycodone (*n* = 9, mean ± SD).

Index	Unit	All patients	Male	Female
AUC_(0 → 24h)_	h·ng/mL	101.06 ± 36.22	111.49 ± 42.56	83.68 ± 15.69
AUC_(0 → ∞)_	h·ng/mL	145.73 ± 67.18	158.20 ± 85.21	124.95 ± 15.43
T_1/2_	h	12.12 ± 4.25	11.06 ± 4.84	13.89 ± 2.98
C_max1_	ng/mL	5.92 ± 2.29	6.78 ± 2.46	4.50 ± 1.22
C_max2_	ng/mL	5.79 ± 1.67	6.40 ± 1.84	4.77 ± 0.64
T_max1_	h	1.06 ± 0.42	0.90 ± 0.22	1.33 ± 0.57
T_max2_	h	7.00 ± 1.85	6.40 ± 2.19	8.00
CL	L/h	60.63 ± 33.90	57.45 ± 42.90	65.92 ± 16.53
V_d_	L	943.51 ± 402.48	729.76 ± 287.77	1299.75 ± 311.08
MRT_(0 → 24h)_	h	9.82 ± 1.18	9.46 ± 1.33	10.42 ± 0.66
MRT_(0 → ∞)_	h	18.94 ± 6.58	17.24 ± 7.38	21.78 ± 4.81
V_ss_	L	1026.80 ± 443.43	793.75 ± 313.42	1415.21 ± 360.12

Comparison of PK parameters between male and female groups showed no significant difference.

## 4 Discussion

This study utilized acetonitrile protein precipitation for plasma sample preparation. This method offers a simpler and faster procedure that requires fewer materials and reagents than solid-phase or liquid–liquid extraction (12, 13), and is well-suited for extracting most small-molecule drugs and their metabolites. As such, it is particularly advantageous for clinical therapeutic drug monitoring and drug–drug interaction studies. Furthermore, acetonitrile protein precipitation helps reduce matrix effects and alleviate ion suppression in mass spectrometry. The specific protocol involved centrifuging the samples after precipitation, collecting 110 μL of the supernatant, diluting it with 40 μL of pure water, and then injecting it for analysis. This dilution step further minimizes matrix effects during drug concentration quantification by HPLC-MS/MS.

During method validation, quantitative ions should be selected based on high signal response and low background noise (14). For oxycodone, two product ions (m/z 256.3 and 241.0) demonstrated favorable intensity. Noroxycodone produced three informative fragments at m/z 284.1, 227.1, and 187.1, while papaverine yielded two prominent ions at m/z 202.2 and 171.3. However, the fragments at m/z 256.3, 284.1, 227.1, and 171.3 exhibited elevated baseline noise and suboptimal peak morphology, precluding their use for quantification. Therefore, m/z 241.0 (oxycodone), m/z 187.1 (noroxycodone), and m/z 202.2 (papaverine) were selected as the quantitative ions.

The selection of column and mobile phase conditions is critical for achieving optimal peak shape and appropriate retention times for the analytes. Published HPLC-MS/MS methods for quantifying oxycodone alone or in combination with its metabolites often employ various columns, including C18 columns from different manufacturers (6, 15), biphenyl columns (16), HSS T3 columns (17), and silica columns (18). Initially, an Agilent Eclipse XDB C18 column (50.0 mm × 2.1 mm, 1.7 μm) was tested in this study. However, the analyte noroxycodone exhibited an asymmetric peak shape and high baseline noise, which interfered with accurate quantification. Attempts to resolve this issue by modifying other parameters proved ineffective. Subsequently, a Phenomenex Kinetex F5 column (3.0 × 100 mm, 2.6 μm) was evaluated. This column demonstrated improvements in peak shape, resolution, and reproducibility, although issues such as peak broadening and tailing persisted, indicating a need for further optimization. Regarding the mobile phase, an initial composition of 2 mmol/L ammonium acetate in water (aqueous phase) and methanol (organic phase) was tested. This combination did not alleviate the observed peak broadening and tailing. The subsequent addition of 0.1% formic acid to both mobile phase components resulted in improved peak width. Nevertheless, the chromatogram for noroxycodone still showed interference. Finally, the gradient elution program was modified. The initial gradient (mobile phase B: 70% at 0 min, 90% from 2–3 min, 70% from 3.1–5 min) was changed to a new program (mobile phase B: 70% at 0 min, 95% from 1.5–2 min, 70% from 2.01–4 min). Under these final conditions, the peaks for oxycodone, noroxycodone, and papaverine (used as an internal standard) were well-resolved from baseline noise, exhibiting symmetric shapes and high intensity. Their respective retention times were 0.93 min, 0.93 min, and 1.08 min. Our research group considers these the optimal chromatographic conditions achieved, satisfactorily meeting the requirements for method validation.

This study included nine HCC patients with confirmed normal preoperative liver function. As shown in Table 5, all liver function biomarkers (ALT, AST, and BIL) were within normal ranges, confirming preserved baseline liver function and excluding potential confounding effects of hepatic impairment on PK parameters. Sex-stratified analysis revealed that liver function remained within normal limits in both groups, indicating that the observed sex-associated PK differences are not attributable to hepatic insufficiency. Therefore, the PK variability observed in this study likely reflects sex differences and perioperative physiological alterations rather than impaired hepatic metabolism. Notably, these sexually dimorphic characteristics are more pronounced in the context of preserved liver function, further supporting our mechanistic interpretation.

With regard to perioperative factors, substantial intraoperative fluid resuscitation and blood loss may expand the volume of distribution of the hydrophilic drug oxycodone (Vss, 223.07 vs. 135.34 L), thereby prolonging its half-life. Meanwhile, surgical stress and hepatic vascular occlusion may impair drug metabolism and clearance, leading to reduced clearance (38.03 vs. 49.8 L/h). Sex-related differences in intraoperative management—higher fluid infusion in males and greater blood loss in females—may partially account for the observed PK variability. Additionally, intergroup differences in age and BMI may also contribute to this variability. Owing to the small sample size, multivariate analysis could not be performed to disentangle these effects. Future large-scale studies are warranted to elucidate the independent contributions of sex, age, and body composition to oxycodone pharmacokinetics.

In addition to the above perioperative factors, significant sex-associated differences in PK parameters were also observed. Further analysis of the PK parameters of oxycodone showed that female patients exhibited higher values for AUC_(0 → 24h)_, AUC_(0 → ∞)_, MRT_(0 → 24h)_, and MRT_(0 → ∞)_ than male patients. This observation is consistent with a lower clearance capacity for oxycodone in females relative to males. As both oxycodone and its metabolites are primarily excreted renally, the lower glomerular filtration rate in females (19) contributes to a slower elimination rate of the drug, resulting in a prolonged retention time in the females.

A gender-associated difference in noroxycodone plasma concentrations was observed, with higher levels detected in male compared to female patients. Given the limited sample size of the present study, these findings should be interpreted as preliminary, and validation in larger cohorts is required. This PK divergence appears to be attributable to testosterone-mediated upregulation of CYP3A4 expression concurrent with inherently reduced CYP2D6 enzymatic activity in males (20). Oxycodone undergoes biotransformation principally via CYP3A4-catalyzed N-demethylation to noroxycodone and CYP2D6-mediated O-demethylation to oxymorphone. Reduced CYP2D6 activity in males could enhance dependence on the CYP3A4 metabolic pathway, potentially favoring noroxycodone formation. In contrast, higher CYP2D6 activity in females may promote oxymorphone generation. Thus, sexually dimorphic expression and function of these CYP450 isoforms may contribute to the observed elevation in noroxycodone exposure among male patients, although this hypothesis warrants further investigation in larger, adequately powered studies.

A comparative analysis of PK parameters revealed substantial differences between the study cohort and literature values reported for healthy volunteers (21). Following intravenous administration of oxycodone hydrochloride injection, healthy subjects exhibited a Vss of 135.34 L, CL of 49.8 L/h, T_1/2_ of 2.61 h, and AUC_(0 → ∞)_ of 70.22 h·ng/mL. In contrast, the corresponding values observed in our perioperative cohort were a Vss of 223.07 L, a CL of 38.03 L/h, a T_1/2_ of 4.8 h, and an AUC_(0 → ∞)_ of 205.59 h·ng/mL, demonstrating significantly enhanced exposure (AUC_(0 → ∞)_), prolonged elimination half-life (T_1/2_), expanded volume of distribution (Vss), and reduced clearance (CL). These PK alterations are likely attributable to pathophysiological changes induced by the perioperative state. Surgical trauma and anesthetic agents elicit systemic inflammatory and stress responses (22), potentially causing transient hepatic dysfunction. Moreover, intraoperative administration of vasoactive compounds (e.g., epinephrine, dopamine) can compromise hepatic and renal perfusion (23), thereby impeding drug elimination. Additional contributing factors include fluid resuscitation-induced hypervolemia, which increases the distribution volume of hydrophilic oxycodone hydrochloride (24). Surgical stress and hepatic resection may also depress plasma protein synthesis (e.g., albumin) (25, 26), reducing protein binding and enhancing tissue penetration of the free drug fraction. Given oxycodone’s moderate plasma protein binding (38–45%), decreased binding capacity would facilitate peripheral distribution, consistent with the observed increases in Vss and T_1/2_. Finally, transient hepatic vascular occlusion during oncologic surgery disrupts normal perfusion patterns, potentially further delaying metabolic clearance and prolonging the elimination phase.

The PK findings of this study have several clinical implications. First, the reduced clearance (38.03 vs. 49.8 L/h) and prolonged half-life (4.8 vs. 2.61 h) in perioperative HCC patients suggest that standard dosing may cause drug accumulation. Therefore, an initial dose reduction of 20–30% with extended intervals is recommended, followed by individualized titration. Second, the markedly increased AUC (205.59 vs. 70.22 h·ng/mL) indicates a higher risk of respiratory depression and excessive sedation. Enhanced monitoring (pulse oximetry, respiratory rate) should continue for 48–72 h postoperatively, with naloxone available. Third, the expanded Vss (223.07 vs. 135.34 L) may delay the achievement of therapeutic concentrations in the central nervous system; a short-acting opioid (e.g., fentanyl) can be used as a bridge until steady state is reached. Finally, the prolonged half-life can facilitate smoother analgesic regimens with reduced peak-to-trough fluctuations, potentially decreasing breakthrough pain and PCA demands. Collectively, these data provide an evidence-based framework for optimizing oxycodone dosing, safety monitoring, and analgesic efficacy in perioperative HCC patients with preserved liver function.

## 5 Conclusion

The present study provides a PK characterization of oxycodone and noroxycodone in perioperative liver cancer patients, revealing distinct gender-dependent metabolic profiles. These observations underscore the necessity for prudent clinical application and individualized dosing of oxycodone hydrochloride injection in this patient population.

## Data Availability

The original contributions presented in the study are included in the article/supplementary material, further inquiries can be directed to the corresponding authors.
